# Persistence of *Bacteroides ovatus* under simulated sunlight irradiation

**DOI:** 10.1186/1471-2180-14-178

**Published:** 2014-07-04

**Authors:** Shengkun Dong, Pei-Ying Hong, Thanh H Nguyen

**Affiliations:** 1 Department of Civil and Environmental Engineering, University of Illinois at Urbana-Champaign, 205 N. Mathews, 3230 Newmark Lab, Urbana, IL 61801, USA; 2 Water Desalination and Reuse Center, Environmental Science and Engineering Program, King Abdullah University of Science and Technology (KAUST), 4700 King Abdullah Boulevard, Thuwal 23955-6900, Saudi Arabia

**Keywords:** *Bacteroides ovatus*, Sunlight irradiation, Fate and persistence, Seawater, Freshwater, PMA-qPCR

## Abstract

**Background:**

*Bacteroides ovatus*, a member of the genus *Bacteroides*, is considered for use in molecular-based methods as a general fecal indicator. However, knowledge on its fate and persistence after a fecal contamination event remains limited. In this study, the persistence of *B. ovatus* was evaluated under simulated sunlight exposure and in conditions similar to freshwater and seawater. By combining propidium monoazide (PMA) treatment and quantitative polymerase chain reaction (qPCR) detection, the decay rates of *B. ovatus* were determined in the presence and absence of exogenous photosensitizers and in salinity up to 39.5 parts per thousand at 27°C.

**Results:**

UVB was found to be important for *B. ovatus* decay, averaging a 4 log_10_ of decay over 6 h of exposure without the presence of extracellular photosensitizers. The addition of NaNO_2_, an exogenous sensitizer producing hydroxyl radicals, did not significantly change the decay rate of *B. ovatus* in both low and high salinity water, while the exogenous sensitizer algae organic matter (AOM) slowed down the decay of *B. ovatus* in low salinity water. At seawater salinity, the decay rate of *B. ovatus* was slower than that in low salinity water, except when both NaNO_2_ and AOM were present.

**Conclusion:**

The results of laboratory experiments suggest that if *B. ovatus* is released into either freshwater or seawater environment in the evening, 50% of it may be intact by the next morning; if it is released at noon, only 50% may be intact after a mere 5 min of full spectrum irradiation on a clear day. This study provides a mechanistic understanding to some of the important environmental relevant factors that influenced the inactivation kinetics of *B. ovatus* in the presence of sunlight irradiation, and would facilitate the use of *B. ovatus* to indicate the occurrence of fecal contamination.

## Background

Earlier studies have reported that the occurrence of gastrointestinal illness in both fresh and coastal waters has been found to correlate statistically with the presence of fecal indicator bacteria (FIB) such as coliforms and enterococci [1,2]. As such, the culture-dependent method to enumerate conventional FIBs is commonly used for detecting fecal contamination. However, this method has always been challenged for various reasons. First, most conventional FIBs can proliferate under natural conditions after being released into the environment, and the detection of these FIBs may overestimate the original contamination level. Second, monitoring the cause of waterborne illness based on conventional FIBs underestimates the actual number of FIBs as it excludes the viable but non-culturable cells [3]. Third, most conventional FIBs exist in feces of a number of warm-blooded animals, which would not allow identification of the host origin [4]. Lastly, FIBs require incubation time of 24-48 h, posing a possibility for people to be exposed to contaminated water before regulatory announcements can be made [5].

Molecular-based approaches like quantitative polymerase chain reaction (qPCR) assays targeting genetic markers for 16S rRNA genes of fecal indicators can be used to overcome the disadvantages of using culture-dependent methods to evaluate fecal contamination. A combination of qPCR and propidium monoazide (PMA) pretreatment has been developed for the *Bacteroides* spp. to distinguish between cells with intact membrane and cells with compromised membrane [6]. The *Bacteroides* spp. have been proposed to replace conventional FIBs [7] as a general indicator for fecal contamination because they are commonly isolated from feces [8] or numerically abundant in feces [9].

Recently, US EPA validated the use of the qPCR approach to quantify the *Bacteroidales* genetic marker for fecal contamination detection [8]. Among the genus *Bacteroides*, *Bacteroides ovatus* is considered for use as a general fecal contamination indicator [10]. However, the different *Bacteroidales* species have been demonstrated to have different durations of persistence, which is likely due to environmental factors and the stability of DNA markers [9]. This different persistence would make it challenging to track the bacterial indicator. Exposure of the *Bacteroidales* (species) cells to sunlight irradiation may be one of the important environmental processes that significantly influence their persistence in surface water [11-13]. DNA marker from the *Bacteroidales* could persist for days or weeks in naturally sunlit seawater and low salinity water environments [14]. Longer persistence of these genetic markers was observed at lower temperatures and higher salinities [15,16]. Natural sunlight was found to have negligible effect on the persistence of these genetic markers [17]. However, variation of natural sunlight irradiation in time and location hinders the application of data obtained with natural sunlight from one location to the other. Accurate correlation between persistence of the *Bacteroidales* cells and sunlight irradiation may, therefore, facilitate the use of these bacterial populations as fecal indicators.

Three fundamental photoinactivation mechanisms have been identified for microorganisms: direct inactivation, indirect exogenous inactivation, and indirect endogenous inactivation. Direct inactivation refers to the direct damage of the cellular components such as the genome and proteins by shorter wavelengths of sunlight [18,19]. This mechanism has been suggested as the dominant mechanism in bacterial photoinactivation in some cases [19]. Indirect endogenous inactivation involves the light absorption by sensitizers that belong to the cell. These sensitizers eventually either pass electrons to other cellular components or create reactive oxygen species that inactivate the cell [19-22]. However, it is difficult to experimentally distinguish direct inactivation and indirect endogenous inactivation [23,24]. Indirect exogenous inactivation usually starts with photo-excitation of the exogenous light sensitizers, such as natural organic matter and nitrate, which react with dissolved oxygen and produce reactive oxygen species such as **·** OH that can oxidize and damage cellular components [20]. Nevertheless, the contribution of each mechanism to the inactivation of each specific bacterium species is still unclear [25]. Study on inactivation mechanisms of *B. ovatus* is particularly lacking.

The objective of this study was therefore to evaluate the decay of *B. ovatus* under conditions similar to both freshwater and coastal seawater, in the presence of controlled simulated sunlight and extracellular algal natural organic matter (AOM) from *Skeletonema costatum*. This algal species was chosen due to its wide distribution globally [26]. The PMA-qPCR approach was used to differentiate and enumerate cells with and without intact membrane. The mechanisms controlling the decay of *B. ovatus* were determined based on the results of experiments, which were designed to answer a number of questions on the effects of sunlight UV irradiation and the roles that exogenous photosensitizers (NO_2_^-^ and AOM), and water salinity would have on the persistence of fecal indicator *B. ovatus* in the water environment (see Additional file 1: Table S1 for a summary of research questions, experimental design, and findings).

## Methods

### *B. ovatus* cultivation

A lyophilized *Bacteroides ovatus* strain ATCC 8483 received from American Type Culture Collection was first suspended in inoculating fluid (Biolog Inc. 72401), followed by inoculation in anaerobic medium [27] with a modified glucose content of 0.5% (w/v). After the cell suspension reached an optical density at 600 nm (OD_600_) of 1, 0.5 ml of the suspension was collected and subsequently inoculated into a serum bottle with 60 ml of anaerobic medium. *B. ovatus* cells were harvested after 14.5 h of incubation at 37°C by centrifugation at 15,557 g for 16 min at 20°C. The pellets were washed three times with 10 mM sterilized phosphate buffer solution at pH 8.2 by sequential centrifugation at 15,557 g before irradiation experiments.

### Extracellular AOM preparation

Bulk microalgae solution of the species *S. costatum* LB 2308 propagated in Erdschreiber’s medium was purchased from The Culture Collection of Algae at the University of Texas at Austin (UTEX). The bulk solution was subject to centrifugation at 2740 g for 10 min, and the supernatant containing organic cell exudates was collected. The supernatant was filtered through a cellulose acetate membrane of 1.2 μm pore size (EPS®, Inc.) to remove algae cells and debris. The filtered supernatant was subjected to dialysis using 3.5 k Dalton MWCO membrane (Thermo Scientific SnakeSkin, 88245) against deionized water. The dialysis concentrate, which is referred to as the extracellular AOM, was harvested when the solution conductivity was below 10 μS/cm. The extracellular AOM after dialysis had total organic carbon (TOC) of 1.94 mg C/L, specific UV absorbance at 254 nm SUVA of 1.03, pH of 6.18, and salinity < 1 parts per thousand at 27°C.

### Experimental setup

Eighty ml glass beakers (Pyrex) were used as microcosms, which were wrapped with black tape (3 M) to prevent the reflected and deteriorated simulated sunlight screened by the beaker from reaching the liquid inside the microcosm. Mixing was facilitated by a Variomag electronic stirrer set at 130 rpm. Seawater temperatures recorded near King Abdullah University of Science and Technology (KAUST) were 20°C in February 2013 and 36°C in May 2013. Therefore, experiments in this temperature range were conducted. Temperature of the reactors was regulated by a water bath set at 27°C. A reactor covered with a piece of aluminum foil was used as the dark control.

Solar exposure was conducted in an Atlas Suntest® XLS + photosimulator (Chicago, IL) equipped with a xenon arc lamp. A 280 nm cutoff filter (Newport, MA) was placed on top of reactors to prevent liquid loss through evaporation. A 320 nm cutoff filter (Newport, MA) was used to determine the significance of UVB for *B. ovatus* inactivation. An ILT950 spectroradiometer (International light technologies, MA) was used to measure the irradiance from 280 to 700 nm inside the solar simulator. The xenon arc lamp was set at an irradiance of 400 Wm^-2^ with a UV special glass filter, which delivers 3248 μW/cm^2^ of UV irradiance received by the reactors. In comparison, the UV irradiance on the University of Illinois campus in Urbana during a clear day at 12 pm on April 22^nd^ was 3899 μW/cm^2^, and on October 21^st^ a total UV irradiance of 2618 μW/cm^2^ was measured at around 2 pm on the KAUST campus during a clear day in Jeddah, Saudi Arabia. To convert the irradiance (μW/cm^2^) to fluence (J/cm^2^), cumulative irradiance over 280-700 nm wavelength for a specified duration of time was calculated.

A final cell concentration of 10^9^-10^10^ cells/ml was used for all solar exposure experiments. For experiments with low salinity and without AOM, 10 mM sterilized phosphate buffer solution (Fisher Scientific Inc.) was prepared to resuspend the washed *B. ovatus* cell pellet to 60 ml. Samples were taken intermittently after the initiation of the exposure to the simulated sunlight. 1.2 ml of aliquot was drawn out of each microcosm and stored at -20°C for a day before DNA extraction using the Ultraclean Soil DNA Isolation Kit (Mo-Bio, CA) following the manufacturer’s instructions. Two batches of samples were collected from a given reactor, one was treated with PMA to determine intact cell count and the other was not. Both batches were subsequently enumerated with qPCR assay. For reactors with AOM, natural organic matter stock solution was added to the beaker, and 500 mM sterilized phosphate buffer (Fisher Scientific Inc.) was used to adjust the pH to 8.2. Ionic strength was then compensated with sodium chloride to 10 mM. For selected experiments, 10 mg/L of NaNO_2_ was used as synthetic sensitizer of **·** OH. Preliminary experiments on *Escherichia coli* to optimize the majority of the workflow were done in triplicates. Experiments done on *B. ovatus* were conducted five times as a result of the power analysis (1–β = 80%).

To mimic the high salinity condition that *B. ovatus* may experience upon being released into the sea, solar exposure experiments were also conducted in artificial seawater, containing 32 g/L of sodium chloride, 0.8 g/L of potassium chloride, 1.3 g/L of calcium chloride, and 6.1 g/L of magnesium chloride. This ionic concentration was selected to match salinity of Red Sea water at 39.5 parts per thousand collected on the KAUST campus [28]. Experiments conducted with this artificial seawater were referred to as high salinity, while experiments conducted with solutions of 10 mM ionic strength were referred to as low salinity.

### Propidium monoazide (PMA) treatment

PMA (Biotium Inc., CA) was used to count cells with intact membrane. This chemical binds to double stranded DNA in both the extracellular environment and in cells with compromised membrane, preventing subsequent qPCR amplification as previously described in other studies [6,17]. 2 mM of PMA stock solution was prepared using 20% DMSO as the solvent and stored in the dark at -20°C until used. For each PMA treatment, 63 μl of the stock solution was added into the 1.2 ml sample to achieve 100 μM of the final PMA concentration. The samples containing PMA were incubated for 5 min on a rotary shaker in the dark, followed by exposure to two 500 W halogen lamps for 6 min at a distance of 20 cm to photoactivate the dye. During the exposure, the samples were placed on top of the aluminum foil with a tray of ice beneath them to prevent samples from overheating.

### Quantitative PCR (qPCR)

Amplification and detection of the targeted 16S rRNA gene of *B. ovatus* were used to enumerate both intact and/or compromised cells. 384-well clear optical reaction plates (Applied Biosystems®, CA) were used in an ABI PRISM 7500 HT real time quantitative PCR machine (Applied Biosystems®, CA). A MicroAmp optical adhesive film (Applied Biosystems®, CA) was used to cover each 384-well clear optical reaction plate to prevent evaporation and contamination. Each 15 μl qPCR reaction mixture contained 2 μl of DNA template and 13 μl of qPCR mastermix. The mastermix consisted of a mixture of Power SYBR Green (Applied Biosystems®, CA), 0.2 μM Bac32F and Bac303R primers (IDT, IA), and molecular biology grade water (Thermo Scientific, MA). Triplicate analysis was done for each sample, and a standard curve was generated with serial dilution of Promega pGEM®-T Easy Vector (Madison, WI) harboring the entire 16S rRNA gene from *B. ovatus.* The thermal cycling parameters include an initial 2 min at 50°C, followed by 10 min at 95°C, and 40 cycles of 15 s at 95°C and 1 min at 60°C. A dissociation cycle of 15 s at 95°C, followed by 15 s at 60°C, ramping up to 95°C for 15 s was done to assess for non-specific amplification. Two samples that contained all the qPCR reagents except for the DNA samples were prepared as negative controls to check for PCR contamination. The detection limit for the Bac32 F and Bac303R primers is 29 copies per reaction, with amplification efficiency for the set of primers ranging from 90-94%.

### Decay rate calculations

*B. ovatus* cells with intact membrane will be referred as “intact cells” and cells with compromised membrane will be referred as “compromised cells” below. Pseudo-first decay model was used to fit the data and *k*_*obs*_, the pseudo-first decay rate constant, was obtained by linear regression fitting. Light screening correction was done on *k*_*obs*_ to account for the light absorption and scattering in the solution [29]. Besides the inactivation rate constant, the half-life of *B. ovatus* decay was also calculated. Paired sample *t*-test was used to determine if the treatment group was significantly different from the dark control group. One-way ANOVA test was done to determine if *k*_*obs*_ or half-life among more than three treatment groups were significantly different from each other. Power 1-β = 80% was used for all statistical tests.

## Results

### Influence of UV irradiation on *B. ovatus* decay

**Figure 1:**
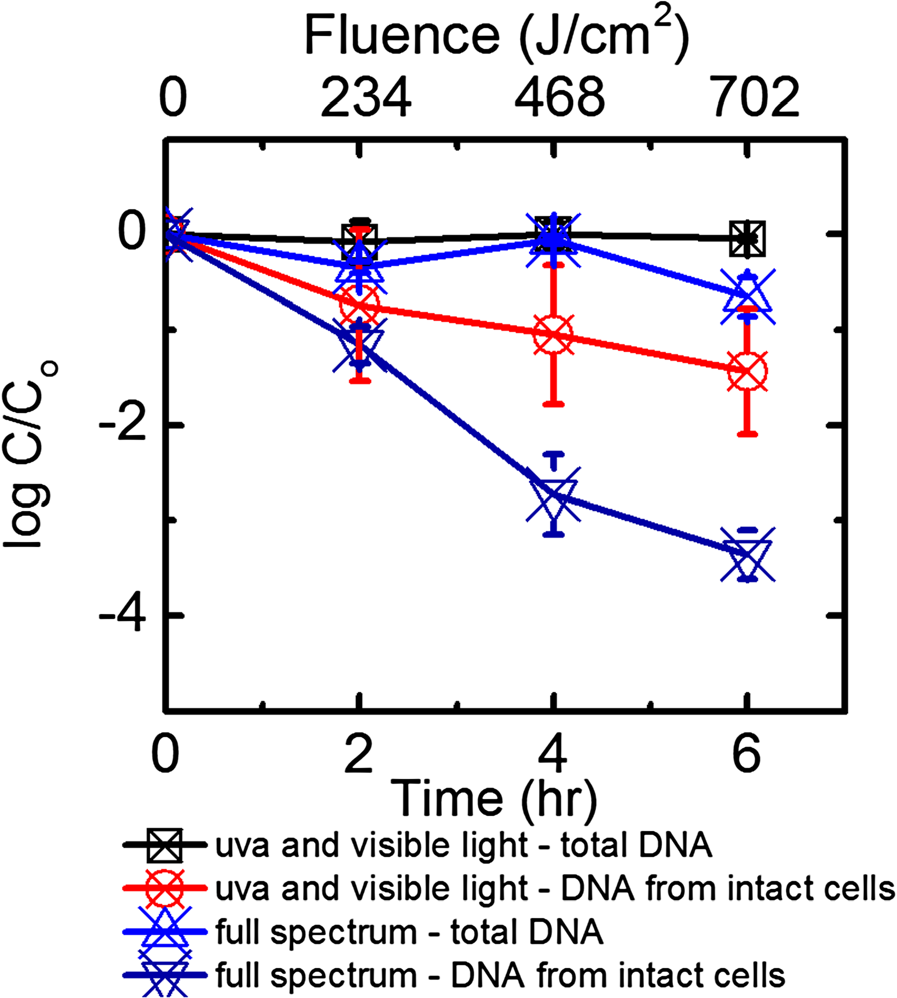
**Comparison of the persistence of *B. ovatus *in low salinity water irradiated by full spectrum simulated sunlight and UVA with visible light.** Error bars correspond to standard deviation of up to 5 replicates. Data were not corrected for light screening.

For the full spectrum irradiation experiment at 27°C comprising UVB, UVA, and visible light exposure, 4 log_10_ of decay was observed over 6 h of experiment, with a pseudo-first decay rate constant (*k*_*obs*_) significantly different from zero (p < 0.05, α = 0.05, Table 1, Figure 1). The total *B. ovatus* cells, including both intact and compromised, decreased 25% over 6 h of exposure to full solar spectrum. When UVB was blocked and *B. ovatus* cells were exposed to 6 h of simulated sunlight containing only UVA and visible light, 1.5 log_10_ of decay was observed. The presence of UVB clearly led to faster *B. ovatus* decay, reflecting in an additional 2.5 log_10_ of decay over 6 h of exposure. As a result, to reveal a more prominent decay trend in subsequent experiments, full spectrum irradiation containing UVB, UVA, and visible light was used. In low salinity water irradiated by full spectrum simulated sunlight, the half-life calculated based on the pseudo-first decay model for intact cells was 0.08 h, or 4.8 min. This half-life is significantly shorter than 9 h, which was determined for cells kept in the dark. For the dark control, no significant growth or decay was observed (Figure 1).

**Table 1:** **Decay rate constants observed for *B. ovatus***

**Condition**	***k***_***obs ***_**in hr**^**-1 **^**for low salinity water (half-life in h)**	***k***_***obs ***_**in hr**^**-1 **^**for high salinity water (half-life in h)**
Phosphate buffer irradiated by UVA and visible light	4.1 ± 2.0	Not available
	(0.21 ± 0.12)	
Phosphate buffer irradiated by full spectrum	9.1 ± 1.0	3.3± 0.4
	(0.08 ± 0.009)	(0.21 ± 0.02)
Phosphate buffer dark control	0.08 ± 0.04	-0.01 ± 0.04
	(9.17 ± 3.28)	(4% increase)*
Phosphate buffer and NaNO_2_ irradiated by full spectrum	6.7 ± 1.7	4.2 ± 0.3
	(0.11 ± 0.02)	(0.17 ± 0.01)
Phosphate buffer and NaNO_2_ dark control	0.06 ± 0.04	0.02 ± 0.01
	(12.84 ± 7.18)	(34.12 ± 17.5)
AOM solution irradiated by full spectrum	2.1 ± 0.3	4.0 ± 1.2
	(0.34 ± 0.05)	(0.19 ± 0.04)
AOM solution dark control	-0.02 ± 0.04	0.04 ± 0.01
	(7% increase)*	(16.18 ± 0.19)
NaNO_2_ AOM solution irradiated by full spectrum	4.7 ± 1.9	7.0 ± 2.0
	(0.17 ± 0.05)	(0.1 ± 0.03)
NaNO_2_ AOM solution dark control	-0.05 ± 0.08	-0.03 ± 0.02
	(36% increase)*	(9% increase)*

*Half-life could not be calculated due to the increase in biomass, therefore the percentage of growth was calculated. Data were corrected for light screening. Kinetic data are also plotted in Additional file 1: Figure S1.

### Influence of exogenous · OH and algae organic matter on *B. ovatus* decay in low salinity water

**Figure 2:**
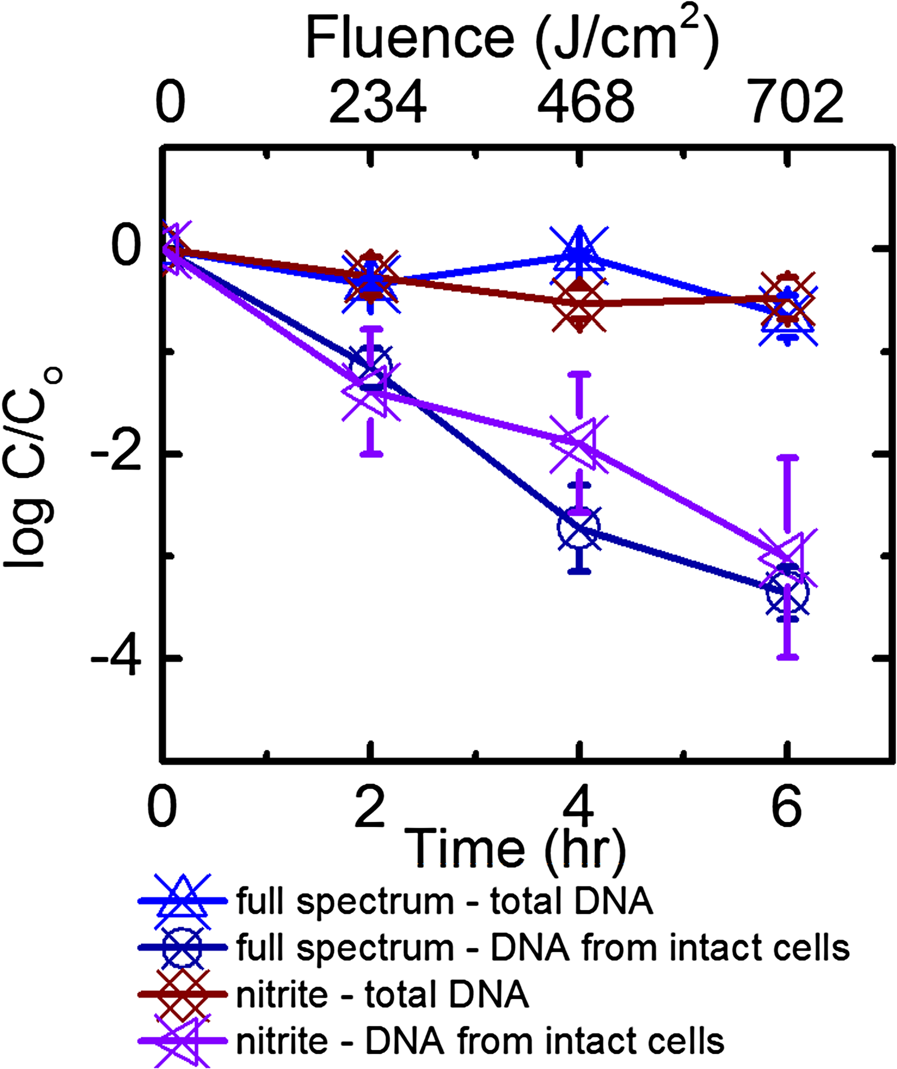
**Comparison of the persistence of *B. ovatus *irradiated by full spectrum simulated sunlight in low salinity water and 0.14 mM NaNO**_**2**_**.** Error bars correspond to standard deviation of up to 5 replicates. Data were not corrected for light screening.

The role of exogenously produced · OH was studied because these highly reactive radicals can non-specifically attack and oxidize cellular components [30]. Production of 0.77 ± 0.08 fM **·** OH in low salinity water was observed from solutions containing 0.14 mM NaNO_2_ exposed to full solar spectrum irradiation at 27°C. The decay rate constants (*k*_*obs*_) measured based on the intact cells were not significantly different for solutions with and without NaNO_2_ in the low salinity water (p > 0.1, α = 0.1, Table 1, Figure 2). These similar *k*_*obs*_ values suggested that the presence of 0.77 ± 0.08 fM **·** OH did not significantly impact the inactivation of *B. ovatus* in low salinity water.

Decay experiments were conducted with a solution containing 2 mg C/L of extracted AOM to simulate some of the actual environmental conditions that *B. ovatus* may be exposed to after being released into the environment. This TOC value was chosen based on the seawater water quality measurement in Saudi Arabia [28]. As shown in Table 1 and Additional file 1: Figure S1a, at 27°C in low salinity water, the presence of AOM slowed down the decay by more than 77% (4.71 ± 1.86 h^-1^ vs.9.09 ± 0.98 h^-1^). This observation is in agreement with lower concentrations of **·** OH observed for solution containing AOM (Table 2). The pseudo-first decay rate constants were higher in solutions with or without NaNO_2_ compared with those obtained in experiments containing both NaNO_2_ and AOM (p < 0.15, α = 0.15, Table 1). These observations indicate that AOM significantly slowed down the *B. ovatus* inactivation.

**Table 2:** **[**^**•**^**OH] for different conditions**

**Experimental conditions**	Hydroxyl radical concentration* (fM)
Low salinity water + NO_2_^-^	0.77 ± 0.08
Low salinity water + AOM	0.09 ± 0.04
Low salinity water + AOM + NO_2_^-^	0.59 ± 0.12
High salinity water + NO_2_^-^	0.36 ± 0.13
High salinity water + AOM	0.02 ± 0.01
High salinity water + AOM + NO_2_^-^	0.42 ± 0.16

*Data were corrected for light screening.

The solution containing AOM has a half-life of 0.34 h, the longest among the data recorded for low salinity irradiated experiments. For the solution containing NaNO_2_, half-life of 0.11 h was significantly shorter than those obtained for solutions containing AOM but not significantly different than those obtained for sensitizer-free solutions (p > 0.05, α = 0.05, Table 1). When solutions were kept in the dark, long half-life values of more than 12 h were observed. In addition, 7% increase in *B. ovatus* biomass was observed for solutions containing AOM kept in the dark.

### Influence of salinity on *B. ovatus* decay

**Figure 3:**
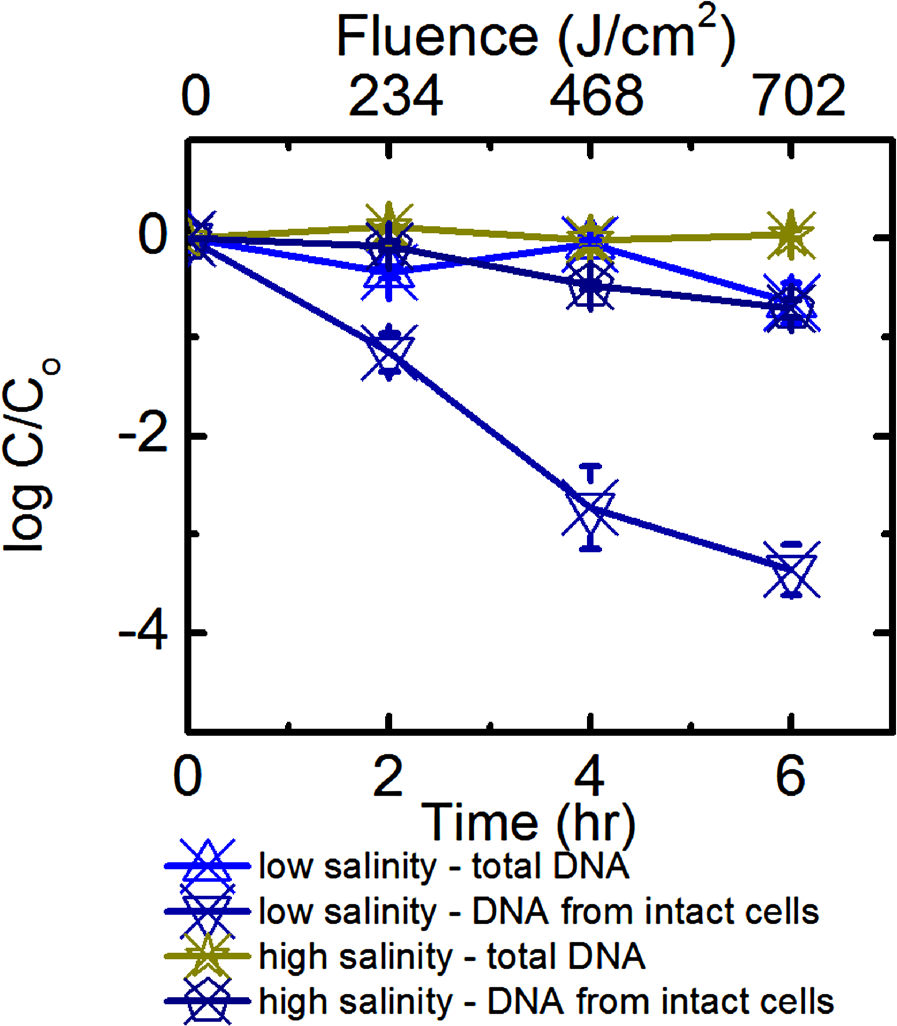
**Comparison of the persistence of *B. ovatus *irradiated by full spectrum simulated sunlight in low salinity and high salinity water.** Error bars correspond to standard deviation of up to 5 replicates. Data were not corrected for light screening.

For experiments without exogenous sensitizers and experiments with only NaNO_2_ as the sensitizer, the decay rate constants of *B. ovatus* in high salinity solutions under full spectrum simulated sunlight were significantly smaller than that at lower salinity (p < 0.05, α = 0.05, Figure 3, Additional file 1: Figure S1b). Less than 1 log_10_ of *B. ovatus* decay was observed over 6 h of exposure, with the total cell count changed insignificantly throughout the experiment. Similar to the findings with low salinity experiments, 0.14 mM NaNO_2_ in AOM-free high salinity solutions did not significantly change *B. ovatus* decay rate constants (p > 0.05, α = 0.05, Table 1). For AOM-containing solutions, high salinity did not lead to a significant decrease in *k*_*obs*_ values (p > 0.05, α = 0.05, Table 1). In both cases, 1 log_10_ decay of intact *B. ovatus* was observed, with the total cell count decreased slightly over time. In solutions containing both 0.14 mM NaNO_2_ and AOM, and only AOM, high salinity led to faster *B. ovatus* decay compared with corresponding low salinity settings (7.01 h^-1^ vs.4.71 h^-1^; 4.03 h^-1^ vs.2.06 h^-1^, Table 1), despite similar **·** OH concentrations (p > 0.05, α = 0.05, Table 2). Mixing 0.14 mM NaNO_2_ and AOM in a high salinity setting yielded an unexpected result, with *B. ovatus* decaying faster than when either 0.14 mM NaNO_2_ or AOM was present alone in the high salinity environment (p < 0.05, α = 0.05, Table 1, Additional file 1: Figure S1c). However, this synergistic effect was not observed for low salinity solutions.

**Figure 4:**
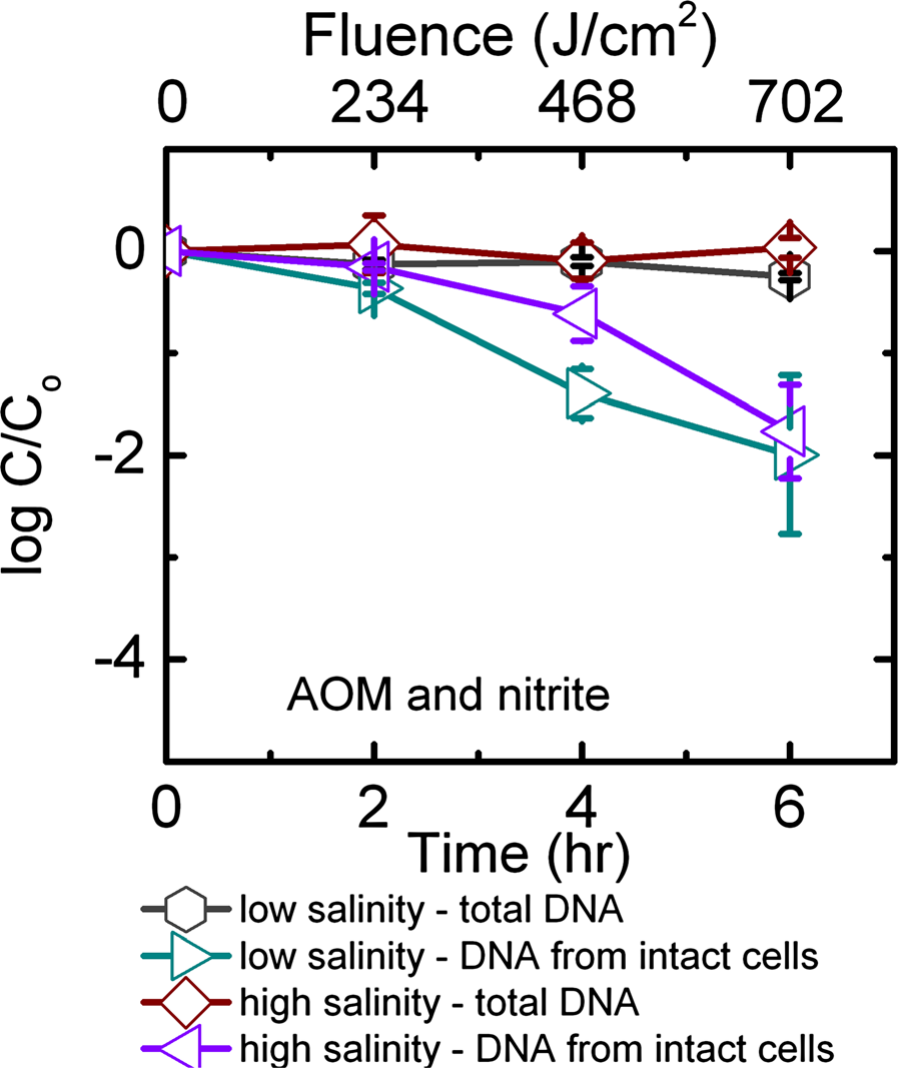
**Persistence of *B. ovatus *irradiated by full spectrum simulated sunlight in artificial seawater containing AOM and 0.14 mM NaNO**_**2**_**, and that in phosphate buffer containing AOM and 0.14 mM NaNO**_**2**_**.** Error bars correspond to standard deviation of up to 5 replicates. Data were not corrected for light screening.

For high salinity solutions irradiated by full spectrum simulated sunlight, a half-life of 0.21 h was recorded, which was longer than that in low salinity solutions (p < 0.05, α = 0.05, Table 1). The half-life values were statistically the same for high salinity solutions with and without NaNO_2_ (p > 0.05, α = 0.05). The presence of both NaNO_2_ and AOM in high salinity solutions led to the observed half-life of 0.1 h, which was significantly shorter than when either NaNO_2_ or AOM was present alone in the high salinity environment (p < 0.05, α = 0.05, Table 1). When both NaNO_2_ and AOM were present, high salinity half-life was also shorter than those obtained from the low salinity (p < 0.05, α = 0.05, Table 1, Figure 4). For all dark controls in high salinity experiments, the decay kinetics of total *B. ovatus* cells had a zero slope (p > 0.05, α = 0.05), indicating that salinity alone did not exert a significant impact on *B. ovatus* persistence in the dark. Irradiation experiments in high salinity water with UVB cut-off was not conducted because UVB irradiation was already identified as an important decay mechanism for *B. ovatus.*

## Discussion

### Mechanisms of *B. ovatus* decay

From the dark control data for low and high salinity conditions, PMA-qPCR did not detect changes in the copy numbers of total cells (i.e., *B. ovatus* with both intact and compromised membrane) and intact cells, suggesting that solution conditions alone did not lead to cell decay or membrane damage. This may be explained by the fact that though *B. ovatus* are strict anaerobes, they possess superoxide dismutase, which helps to fight oxidative stress to a certain level [31,32]. The oxidative stress created by dissolved oxygen for dark controls happened to be within the capacity of superoxide dismutase, enabling *B. ovatus* to remain intact in the dark control. However, for irradiated samples, total cells decayed much less than intact cells, suggesting that irradiation damaged cell membranes. This is in agreement with past studies that reported damage to viral protein capsid by medium-pressure UV and endogenous inactivation of *E. coli* by UVB [33,34].

In the 6 h time frame of this study, more than 25% decay of the total amount of DNA was observed as a result of UVB irradiation in the low salinity settings. This observation agrees with a previous study, where the abundance of total DNA extracted from cells decreased over time upon solar exposure in freshwater microcosms [17]. Following the method to interpret UVC inactivation of antibiotic resistant bacteria developed by McKinney and Pruden [35], the pyrimidine dimers for the whole genome of *B. ovatus* (GenBank accession NZ_DS264553-DS264584) constructed from 32 scaffolds were counted with a MATLAB program and the results were presented in Table 3. The percentage of thymine dimers per *B. ovatus* genome size was 9.5%, which was lower than that of methicillin-resistant *Staphylococcus aureus*, vancomycin-resistant enterococci, and *E. coli* SMS-3-5. Compared to these bacterial species, *B. ovatus* may be more susceptible to UV irradiation than these three bacterial species because of low percentage of thymine dimers per *B. ovatus* genome size.

**Table 3:** **Pyrimidine dimer counts for *B. ovatus *** **and antibiotic resistant bacteria (ARBs)**

		**Dimers (count)**					**Dimers/amplicon length (%)**					**Dimers/total dimers (%)**			
**Gene name**	**Genome size (bp)**	**TT**	**CC**	**TC**	**CT**	**Total**	**TT**	**CC**	**TC**	**CT**	**Total**	**TT**	**CC**	**TC**	**CT**
B. ovatus*	6465369	612293	298804	413170	351734	1676001	9.5	4.6	6.4	5.4	26	36.5	17.8	24.7	21
Data on ARBs as a comparison from McKinney and Pruden, 2012															
MRSA	2872769	706446	145879	302081	766117	1431985	24.6	5.08	10.5	9.66	49.8	49.3	10.2	21.1	19.4
VRE	2826716	638915	199341	373437	318366	1530059	22.6	7.05	13.2	11.3	54.1	41.8	13.0	24.4	20.8
*E. coli* SMS-3-5	5068389	746734	589024	584759	517931	2438448	14.7	11.6	11.5	10.2	48.1	30.6	24.2	24.0	21.2
*P. aeruginosa* 01	6264404	376612	1171802	766117	707725	3022256	6.01	18.7	12.2	11.3	48.2	12.5	38.8	25.3	23.4

*Genome constructed from 32 scaffolds (Accession: NZ_DS264553-DS264584).

MRSA = methicillin-resistant *Staphylococcus aureus*.

VRE = vancomycin-resistant *enterococci*.

In addition, bacteria with larger genome sizes were observed to be more susceptible to UV damage, presumably because larger genomes offered more base per sites for UV damage [35]. The size of the *B. ovatus* constructed from 32 scaffolds was approximately 6.5 Mbp, and was larger than the genome of *Pseudomonas aeruginosa* 01 (i.e., 6.3 Mbp) that was evaluated by McKinney and Pruden [35]. *P. aeruginosa* 01 had the largest genome compared to methicillin-resistant *S. aureus*, vancomycin-resistant *enterococci*, and *E. coli* SMS-3-5, and was determined to be most susceptible to UV damage. As such, it is likely that *B. ovatus* is equally, if not more, susceptible to UV radiation than *P. aeruginosa* 01.

The statistically similar inactivation rate constants with and without extracellular **·** OH produced by NaNO_2_, both in low or high salinity settings, suggest that exogenous inactivation was much less significant than endogenous inactivation mechanism for *B. ovatus*. Specifically, the presence of exogenous **·** OH produced by 0.14 mM of NaNO_2_ did not promote significant *B. ovatus* decay. In surface water, nitrite is usually converted to nitrate during nitrification. Only in places with incomplete nitrification can nitrite concentrations of up to 66 μM be found [36]. In seawater, the concentration of nitrite has been found to be even lower, and accounts for a concentration of 10.5 ± 2 nM [37]. Thus, it is likely that the persistence of *B. ovatus* in water bodies would not be controlled by environmentally-relevant concentration of **·** OH produced by nitrite or nitrate.

The presence of AOM in the extracellular environment slowed down the decay of *B. ovatus* in the low salinity setting, suggesting that AOM may protect *B. ovatus* from sunlight irradiation. When *B. ovatus* is discharged into the low salinity water environment where AOM is also present on a sunny day, 50% of the total intact cells will be lost within 20 min, persisting longer than those conditions without AOM (i.e., 5 min). Our observations suggest that in pockets of freshwater directly exposed to discharge from wastewater treatment plants, it is anticipated that the AOM would be higher than 1.94 mg C/L and would facilitate a longer persistence of *B. ovatus*. A possible explanation to account for a longer persistence of *B. ovatus* in low salinity water with AOM may be due to the partial coverage of the cell surface where the endogenous sensitizers are located by AOM. The solar irradiation that reaches the cell to produce endogenous reactive oxygen species could be attenuated by this layer of AOM, resulting in less endogenously produced **·** OH that can inactivate the cells.

In contrast, the presence of AOM in extracellular environment did not slow down the decay of *B. ovatus* in high salinity water. This can possibly be accounted for by the partial scavenging of endogenously produced or exogenously produced **·** OH in the presence of chloride ions [38]. Therefore, when *B. ovatus* is released into seawater with AOM, *B. ovatus* cells have a half-life of 11.4 min, which is essentially the same as the half-life for the high salinity water without AOM (i.e., 12.6 min). A synergistic effect of AOM, **·** OH, and high salt concentration led to a faster decay of *B. ovatus* in high salinity environment than that without NaNO_2_ (p < 0.05, α = 0.05, Table 1). However, this synergistic effect only translates to a difference of 4.2 min in the half-life of *B. ovatus*, which might not be apparent when performing actual fecal monitoring efforts on-site.

### Implication for use as fecal indicator

Sunlight irradiation, in particular UVB, played an important role in the endogenous inactivation of intact *B. ovatus* cells. To illustrate, the half-life of *B. ovatus* ranged from 9 to 13 h in low salinity and 16 to 34 h in high salinity solutions when sunlight irradiation was absent. This meant that if *B. ovatus* were to be released into either fresh or seawater environment in the evening or night-time, at least 50% of the cells would persist and remain detected by the next morning even though the contamination event may not be recent. On the contrary, if *B. ovatus* were to be released into fresh or seawater environment in the daytime, the cells would only persist for up to 6 h, and a positive detection of *B. ovatus* would be indicative of the occurrence of fresh fecal contamination. Our observations, therefore, provide a possible explanation to account for the different test outcomes when monitoring the conventional fecal indicators at different times of the day [39,40].

At the same fluence value exerted by the solar simulator, *B. ovatus* cells with intact membrane decayed much faster than the *B. ovatus* total DNA (Figure 3). The half-life of total DNA is from 40-100 times longer than that of the intact cells. This observation agrees with a previous study by Walters et al. [14], showing that a *Bacteroidales* human-specific DNA marker decayed slower than cells of the members in the *Bacteroidales* upon exposure to natural sunlight. This would imply that the currently proposed method by US-EPA to determine fecal pollution, which is based on the qPCR enumeration of the total DNA of the *Bacteroidales*, might still be limited in its efficacy to determine recent fecal contamination events.

A limitation of this study is that the high decay rates of *B. ovatus* may be attributed to the constant exposure to irradiance from the solar simulator, and may not represent the diurnal sunlight exposure in the natural environment. To illustrate, an earlier study did not observe a significant impact on the persistence of the species in the *Bacteroidales* in seawater under diurnal sunlight [4]. In the natural environment, temperature fluctuations, insufficient solar radiation transmittance, as well as possible absence of solar UVB due to the possible overcast weather may occur and prolong the persistence of the members in the *Bacteroidales*. Regardless, this study yielded similar *B. ovatus* decay trend at the early stage of the solar irradiation experiment (<6 h) as that obtained from a microcosm exposed to diurnal sunlight [17]. This suggests that the decay kinetic data obtained in this study remains useful in predicting the persistence of intact *B. ovatus* cell biomass after its release into the water environment. The decay kinetic data would allow subsequent calculation of the original amount of biomass upon release, and therefore assist in using this bacterial species as a general fecal indicator.

## Conclusions

In this study, solar UVB was important for the persistence of intact *B. ovatus* in both low and high salinity water. *B. ovatus* had longer half-life in high salinity water than in low salinity water under the same conditions with full spectrum UV radiation (0.21 h vs. 0.08 h) and in the dark (34.12 h vs. 12. 84 h). In low salinity water, the half-life of *B. ovatus* under simulated full spectrum sunlight was enhanced by 4-fold when 2 mg C/L AOM was present. Extracellular **·** OH produced by 0.14 mM NaNO_2_ did not have an observable effect on *B. ovatus* persistence. In high salinity water, neither AOM nor extracellular **·** OH had significant effect on *B. ovatus* persistence. However, the synergistic effect of AOM and extracellular **·** OH produced by NaNO_2_ led to the lowest *B. ovatus* half-life of 0.1 h. Without exposure to sunlight, *B. ovatus* half-life ranged from 9 h to 34 h compared with 5 min to 13 min half-life of *B. ovatus* exposed to sunlight, depending on the solution that *B. ovatus* was present in. DNA degradation for *B. ovatus* under full spectrum sunlight irradiation was also observed, although the half-life of the total *B. ovatus* DNA is more than 100-fold longer than that of the viable cells. The decay kinetic data reported in this study would help predict concentrations of *B. ovatus* cells or the DNA after their release into the water environment, therefore assisting in the use of this bacterium as a general fecal indicator. However, extrapolation to real environments has to be made carefully as a number of parameters, such as water depth, turbidity, biological interactions, were not considered in this study, and these parameters may also have an effect on the persistence of *B. ovatus*.

## Competing interests

The authors declare that they have no competing interests.

## Authors’ contributions

PYH and THN conceived the study. SD performed the experiments, data analysis, and statistical analysis. SD, PYH, and THN participated in writing the manuscript. All authors read and approved the final manuscript.

## Supplementary Material

Additional file 1: Table S1 Experimental design, research questions and findings. **Figure S1. (a)** Comparison of the persistence of *B. ovatus* irradiated by full spectrum simulated sunlight in low salinity water with and without AOM. **(b)** Comparison of the persistence of *B. ovatus* irradiated by full spectrum simulated sunlight in low salinity water and high salinity water with 0.14 mM NaNO_2_. **(c)** Comparison of the persistence of *B. ovatus* in artificial seawater irradiated by full spectrum simulated sunlight containing AOM and 0.14 mM NaNO_2_ with AOM. Error bars correspond to standard deviation of up to 5 replicates. Data were not corrected for light screening. Click here for file
