# Supplementary material for: Persistence of Bacteroides ovatus under simulated sunlight irradiation
Source: BMC Microbiol. 2014 Jul 4;14:178. doi: 10.1186/1471-2180-14-178 (PMC4099502; doi:10.1186/1471-2180-14-178)
Supplement: Additional file 1: Table S1 — Experimental design, research questions and findings. Figure S1. (a) Comparison of the persistence of B. ovatus irradiated by full spectrum simulated sunlight in low salinity water with and without AOM. (b) Comparison of the persistence of B. ovatus irradiated by full spectrum simulated sunlight in low salinity water and high salinity water with 0.14 mM NaNO2. (c) Comparison of the persistence of B. ovatus in artificial seawater irradiated by full spectrum simulated sunlight containing AOM and 0.14 mM NaNO2 with AOM. Error bars correspond to standard deviation of up to 5 replicates. Data were not corrected for light screening. [file 1471-2180-14-178-S1.docx]

**Additional file**

Table S1. Experimental design, research questions and findings.


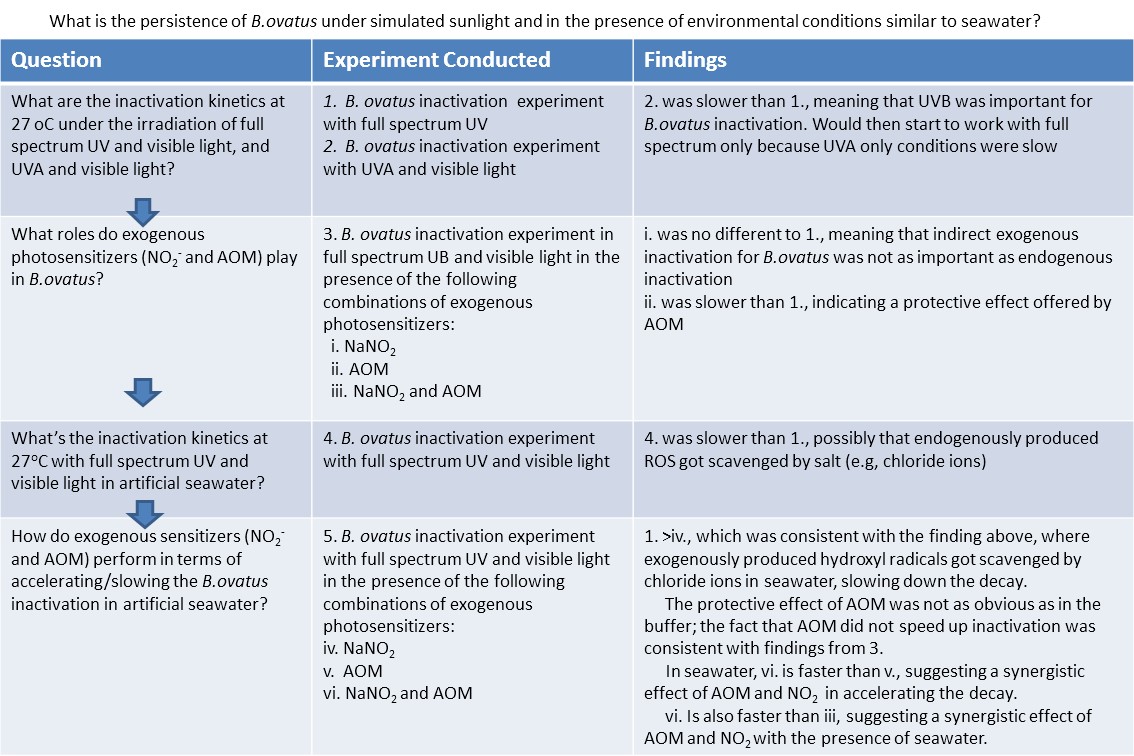









Figure S1 **(a)** Comparison of the persistence of *B.ovatus* irradiated by full spectrum simulated sunlight in low salinity water with and without AOM. **(b)** Comparison of the persistence of *B.ovatus* irradiated by full spectrum simulated sunlight in low salinity water and high salinity water with 0.14mM NaNO_2_. **(c)** Comparison of the persistence of *B.ovatus* in artificial seawater irradiated by full spectrum simulated sunlight containing AOM and 0.14mM NaNO_2_ with AOM. Error bars correspond to standard deviation of up to 5 replicates. Data were not corrected for light screening
